# What drives grassland‐forest boundaries? Assessing fire and frost effects on tree seedling survival and architecture

**DOI:** 10.1002/ece3.6730

**Published:** 2020-09-20

**Authors:** Monique Botha, Sally Archibald, Michelle Greve

**Affiliations:** ^1^ Centre for African Ecology School of Animal, Plant and Environmental Sciences University of the Witwatersrand Johannesburg South Africa; ^2^ Department of Plant and Soil Sciences University of Pretoria Pretoria South Africa

**Keywords:** architectural traits, demographic bottleneck, fire adaptation, frost adaptation, sapling, savanna, survival strategy

## Abstract

1. Fire and frost represent two major hurdles for the persistence of trees in open grassy biomes and have both been proposed as drivers of grassland‐forest boundaries in Africa.
2. We assess the response of young tree seedlings, which represent a vulnerable stage in tree recruitment, to traumatic fire and frost disturbances.
3. In a greenhouse experiment, we investigated how seedling traits predicted survival and resprouting ability in response to fire versus frost; we characterized survival strategies of seedlings in response to the two disturbances, and we documented how the architecture of surviving seedlings is affected by fire versus frost injury.
4. Survival rates were similar under both treatments. However, different species displayed different levels of sensitivity to fire and frost. Seedling survival was higher for older seedlings and seedlings with more basal leaves. Survivors of a fire event lost more biomass than the survivors of a frost event. However, the architecture of recovered fire‐ and frost‐treated seedlings was mostly similar. Seedlings that recovered from fire and frost treatments were often shorter than those that had not been exposed to any disturbance, with multiple thin branches, which may increase vulnerability to the next frost or fire event.
5. *Synthesis*. Fire caused more severe aboveground damage compared with a single frost event, suggesting that fire is an important driver of tree distribution in these open grassland systems. However, the impact of repeated frost events may be equally severe and needs to be investigated. Also, woody species composition may be influenced by phenomena that affect the timing and frequency of seedling exposure to damage, as mortality was found to be dependent on seedling age. Therefore, changes in fire regime and climate are likely to result in changes in the composition and the structure of the woody components of these systems.

## Graphical abstract

Fire caused more severe aboveground damage compared to frost, suggesting that trees in these open grassland systems may be subjected to a seedling release bottleneck maintained by fire. However, the woody species composition will almost certainly be influenced by phenomena that affect the timing and frequency of seedling exposure to damage, as mortality was found to be dependent on seedling age. Therefore, changes in fire regime and climate (esp. changes that bring about less frost and reduced fire intensity and frequency) are likely to result in changes in the composition and the structure of the woody components of these systems.

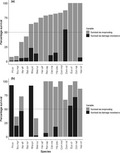

## 1 INTRODUCTION

Open grasslands have long been underappreciated for their biodiversity and ecosystem services (Carbutt, Henwood, & Gilfedder, 2017) and many are under threat due to pressures such as agriculture, urbanization and bush encroachment (the increase of indigenous woody cover in open grassy systems) (Hoekstra, Boucher, Ricketts, & Roberts, 2005; Neke & Du Plessis, 2004; O'Connor & Kuyler, 2009; Skowno et al., 2017). Often mistaken for degraded woodlands and forests, grasslands have been underrepresented in conservation networks, and, more recently, have been targeted as potential sites for forest “rehabilitation” by forestation (Veldman et al., 2015), even though they are often ancient systems (predating anthropogenic activities) and harbor a rich diversity of species (Bond, 2016; Mucina et al., 2006).

**FIGURE 1:**
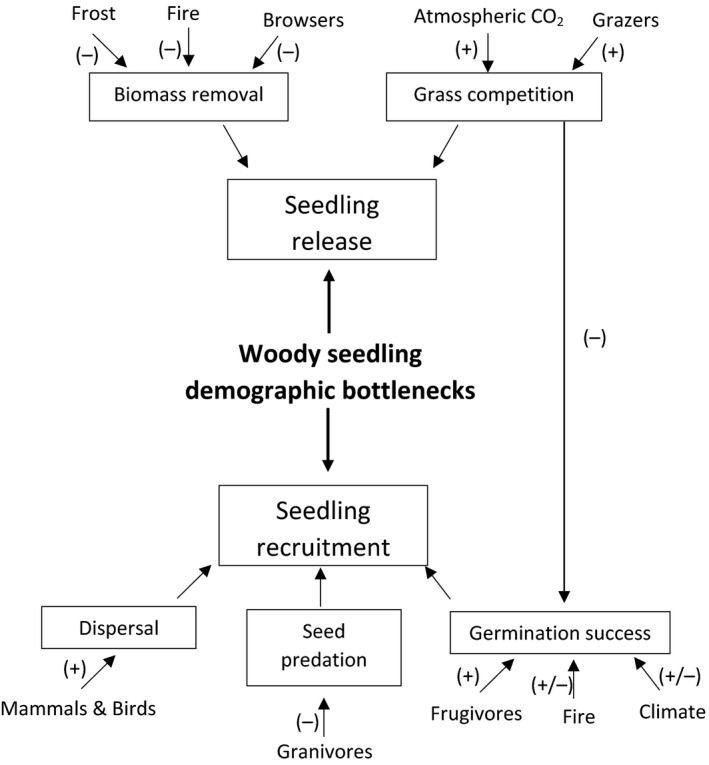
Simplified diagram summarizing some of the main demographic bottlenecks for seedling establishment in open grassy systems. Limiting factors for seedling establishment may act by either preventing seedling recruitment (through failure to disperse or germinate and by seed predation) or by preventing seedling release (seedlings are able to recruit but fail to reach escape height due to grass competition or top‐kill events). Animals, fire, and abiotic environmental conditions (temperature, rainfall, and atmospheric CO_2_ concentration) may ultimately have positive (+) or negative (−) effects on seedling recruitment or release. Adapted from Midgley and Bond (2001)

Despite extensive research (e.g., Higgins, Bond, & Trollope, 2000; Mills, Rogers, Stalmans, & Witkowski, 2006; Scholes & Archer, 1997), there is still no real consensus about what determines the boundaries between grassland and wooded (savanna or forest) vegetation due to the complexity and interactive effects of the various factors affecting these systems. Demographic bottlenecks may be created by several factors that ultimately limit the presence of trees in grasslands (Midgley & Bond, 2001) (Figure 1).

Winter frost has been proposed as a major limiting factor for tree establishment in grasslands. Frost may be especially relevant in African grasslands, since African trees are of tropical or subtropical origin and are generally frost sensitive (Bannister & Lord, 2006; Bredenkamp, Spada, & Kazmierczak, 2002). Paleontological evidence suggests that changes from warm interglacial phases to cooler glacial maxima were mirrored by shifts from woodland savanna to open grassland in Africa (Partridge et al., 1993; Scott, 1982). Elsewhere, frost events prevent tree recruitment by killing saplings or maintaining them in a suppressed state in “frost traps” by repeated annual top kill (Finckh, Revermann, & Aidar, 2016; Holdo, 2007; Wakeling, Cramer, & Bond, 2012; Whitecross, Archibald, & Witkowski, 2012).

Fire may also play an essential role in excluding trees from grasslands since it is a common disturbance in open grasslands (Bond, Midgley, Woodward, Hoffman, & Cowling, 2003; Wakeling et al., 2012). Experimental work illustrates noticeable increases in woody biomass and cover with the exclusion of fire (Bond & Keeley, 2005; De Villiers & O'Connor, 2011; Pinheiro, Azevedo, & Monteiro, 2010; Swaine, Hawthorne, & Orgle, 1992). Similar to frost, fire may kill juvenile trees, or keep them suppressed in fire traps to the advantage of grasses, which are better adapted to survive top kill (Gignoux, Lahoreau, Julliard, & Barot, 2009; Higgins et al., 2000). Where fire keeps woody cover low enough to prevent grass exclusion through light competition, grasses can be effective competitors with trees for below‐ground resources, especially at the juvenile stages (Morrison, Holdo, Rugemalila, Nzunda, & Anderson, 2019).

Whether fire or frost represents the major limiting factor for tree recruitment is widely debated (e.g., Finckh et al., 2016; Maurin et al., 2014), and it is often difficult to distinguish between the effects of fire and frost, since both often occur together in open grassy systems. Both mostly cause top kill (i.e., damage to aboveground buds and shoots) in small seedlings, and little damage to underground structures below 10 cm (Finckh et al., 2016). Fire may damage plants by canopy scorching, by causing cambium necrosis or by phloem death (ringbarking) (Midgley, Lawes, & Chamaillé‐Jammes, 2010). However, recent evidence suggests that hydraulic system failure (xylem damage) caused by extreme heat spikes may be a more common cause of plant death (Midgley, Kruger, & Skelton, 2011; West, Nel, Bond, & Midgley, 2016). Hydraulic failure is also typical of frost damage (Midgley et al., 2010), resulting from ice crystal formation and cavitation of the xylem sap due to repeated freeze and thaw events (Dickison, 2000). Although the outcomes of fire and frost damage for plants are similar (Midgley et al., 2010), the physiological stresses caused by extreme heat and cold may in fact be very different (Dickison, 2000), and it may be expected that different adaptive mechanisms apply to these thermal stressors.

Woody species may adopt different strategies to survive fire and frost. One way is to avoid damage to aboveground structures through specialized protective measures. These “disturbance resistor” species may avoid damage by rapid growth to reach escape height (Balfour & Midgley, 2006; Bond, 2008; Burrows et al., 2008; Higgins et al., 2000), by producing a thicker stem to prevent hydraulic xylem failure (Michaletz, Johnson, & Tyree, 2012; Midgley et al., 2011) or by producing thicker bark that protects the internal cambium and bud meristems (Gignoux, Clobert, & Menaut, 1997; Hoffmann et al., 2009; Midgley et al., 2010; Waters, Burrows, & Harper, 2010). Alternatively, “disturbance tolerators” do not invest in specialized defense mechanisms to avoid damage, but have the ability to resprout from remaining structures after top kill (Archibald, Hempson, & Lehmann, 2019; Clarke et al., 2013; Gignoux et al., 1997). Resprouting ability is dependent on the development, maintenance, and protection of a viable bud bank (Clarke et al., 2013) and belowground reserves (Gignoux et al., 2009; Tomlinson et al., 2012).

Vast areas of African savannas are regularly disturbed by fire and frost, affecting vegetation composition and structure (Bond & Keeley, 2005; Bond et al., 2003). Investigating trait responses of woody seedlings to fire and frost events in this context is essential, as it gives an indication of how different trait complexes may provide advantages to surviving these two different disturbances. This is also important, considering recent vegetation shifts toward increased woody biomass in previously grass‐dominated areas, which have partially been put down to decreased fire frequency and intensity (Bond et al., 2003; Trollope, Trollope, & Hartnett, 2002) and fewer frost events (O'Connor, Puttick, & Hoffman, 2014; Wakeling et al., 2012), and pose a major threat to grassland biodiversity and rangeland productivity (Devine, McDonald, Quaife, & Maclean, 2017; Eldridge et al., 2011; O'Connor & Crow, 1999; O'Connor et al., 2014). Mitigating unwanted changes in tree:grass ratios due to anthropogenic global change requires a good understanding of the factors constraining woody cover at all demographic stages (Ward, 2005).

Survival strategies of seedlings may be different to those of mature trees because of their small size and the lack of protective structures such as thick bark, which may only develop at a later stage of their life cycle. It is important to consider the adaptations of seedlings, given that seedling germination and establishment probably represent a significant bottleneck in tree persistence in many grassland systems (Higgins et al., 2000; Jeltsch, Milton, Dean, Van Rooyen, & Moloney, 1998; Sankaran, Ratnam, & Hanan, 2004; Van Wijk & Rodriguez‐Iturbe, 2002).

The aim of this study was to compare the effects of fire and frost on the seedlings of a selection of woody species during their first growth year. Our specific objectives were to (a) compare survival rates between fire and frost treatments and determine which traits predict seedling survival, (b) explore which traits predict survival strategy (i.e., which traits aid resistance or tolerance), (c) determine the extent of stem regrowth (recovery) after fire and frost, and (d) assess how architectural traits of surviving seedlings compare between fire and frost treatments. We provide one of the first studies on trait related responses to traumatic disturbances at the young seedling stage of African woody species. This may ultimately enable predictions of species composition and distribution across open grassy systems under current conditions and in a changing world.

## 2 METHODS

### 2.1 Experimental design

Nineteen woody species representing a range of habitat preferences were selected (Table S1). These species are all native to one geographic area, namely Buffelskloof Private Nature Reserve (BKNR) (25°17′53.6″S 30°30′31.7″E) in Mpumalanga, South Africa. This reserve supports patches of grassland, savanna, and forest and is currently experiencing encroachment into the open grassy areas (Haddad, 2011). The area receives moderate frost, estimated to range between 9 and 21 mean frost days per annum (Mucina et al., 2006). Fire represents an essential part of the BKNR management strategy. However, because of the inherent risk of fires spreading to adjacent plantations, management block‐burns are conducted only within the timeframe of fire belt burning in forestry areas, namely in autumn or early winter, before any significant frost. (J.E Burrows pers. comm. 2018).

Seeds for each species were ordered from a seed company (Silverhill Seeds), collected in BKNR, or collected from trees in garden cultivation. Seeds were sown in plastic seedling trays filled with standard nursery seedling soil mix early in the growing season (23 October 2017). Seedling trays were placed under 40% black shade netting. The photosynthetically active radiation (PAR) measured on a clear day at midday (12:17 p.m. on 1 March 2018) with a ceptometer (Accu‐PAR LP‐80, Decagon Devices) at seeding tray height averaged 770.67 µmol m^2^ s^−1^ (*SE* = 27.21) compared with 1,655.67 µmol m^2^ s^−1^ (*SE* = 15.95) taken in full sun just outside the shade‐net house. Germinations were recorded every second day over 7 months. Within a week of germinating, newly germinated seedlings were transferred to 3.57 L soil bags filled with a growth medium composed of a 1:1:1 ratio of topsoil, compost, and river sand.

At 74 days, seedlings were transferred to a plot covered by 40% white shade netting. The average PAR value taken at midday (12:20 p.m. on 1 March 2018) at soil bag height was 1,287 µmol m^2^ s^−1^ (*SE* = 13.91) compared with 1,655.67 µmol m^2^ s^−1^ outside the shade‐net house. For each species, up to 15 individuals (Table S2) were assigned to each treatment, although the number of available trees was limited by germination failures and early seedling mortality. Assignment was done to ensure similar‐aged trees were assigned to each treatment.

On 3 May 2018, an experimental fire was applied to seedlings assigned to the fire treatment. The timing of the fire (May) was similar to management fires conducted at BKNR. The day was cool, clear, and with low wind speeds. Seedlings in bags were transferred to a level, open field, and packed in a rectangular 3 m × 6 m soil trench, approximately 30 cm deep. The seedlings were spaced ±30 cm apart. The area between the bags in the trench was then filled up with soil so that the tops of the soil bags were level with the soil surface. A uniform fuel bed of dried grass (approximately 30 cm thick) was lightly strewn around the trees, taking care that the fuel bed had sufficient aeration to prevent smoldering. The fuel load based on the grass biomass in 0.5 m^2^ was estimated at 1,708.08 kg/ha. The fuel bed was extended approximately 1 m upwind of the plot to enable the fire to gain momentum before reaching the first seedlings. A head fire (moving with the wind) was then set in a single line along the length of the plot and allowed to burn through the designated area. Directly after the burn, scorch height (height of highest scorched foliage) was measured for a random selection of seedlings in different positions throughout the plot to give an indication of flame height. The seedling bags were dug out and transported back to the nursery the next day.

The frost treatment was applied to assigned seedlings from 16 to 17 July 2018. Seedlings were transported in a closed vehicle to Lanseria (25°56′13.5″S, 27°52′06.6″E), where they were packed in an open site next to a river, with spacing between individual trees of approximately 50 cm. To insulate the roots of the seedlings, each soil bag was placed in a larger (22 cm diameter) plastic pot and the space between the plastic bag and the pot stuffed with dried grass. Maxim temperature data loggers (iButtons) were placed at different positions throughout the demarcated plot (center and periphery) to monitor soil and air temperatures. Air temperature loggers were placed on stands between the trees, with four at 90 cm and ten at 60 cm above the ground. Ten soil temperature loggers were placed inside the soil bags, between the soil and the edge of the bag, approximately 10 cm below the soil surface. Temperature was recorded at 1‐min intervals and 0.0625°C resolution. The seedlings were left out for two nights to ensure that they experience the natural temperature fluctuations associated with a natural frost event. Upon completion of the treatment, the seedlings were transported back to their original position in the nursery.

The control (no treatment) trees remained under 40% white shade netting for the duration of the experiment.

All seedlings were continually watered during the course of all the treatments by overhead sprinklers. This was done throughout the dry season as seedlings of some species were particularly vulnerable to drought stress in the soil bags due to the low soil volume. Watering was also adapted according to the conditions in the nursery to prevent overwatering.

### 2.2 Trait measurements

Baseline trait measurements of all the seedlings were taken 1 day before each treatment was applied (Table 1). (Baseline measures for frost‐ and fire‐treated individuals took place on different days because these two treatments happened on different days). Additionally, these, and some additional traits, were measured on the same seedlings 2 months after treatment, and again on 1 November 2018 at the end of the experiment (Table 1).

**TABLE 1:** Summary and methodology of the traits measured, and calculated, for tree seedlings

Trait	Data format	Method	Hypothesized predictor contribution to survival	Baseline	2 weeks after treatment	2 months after treatment	Final
Survival	Categorical (alive/dead)	Recorded as dead if no visible live tissue could be observed	NA	Not used	Not measured	Not used	**Response**
Surviving green material	Categorical (present/absent)	Presence or absence of green leaves and active buds	NA	Not measured	**Response**	Not used	Not used
Survival strategy	Categorical (tolerator/resistor)	Classification of live individuals based on presence or absence of green leaves and active buds 2 weeks after treatment. Presence of green material = Resistor; Absence of green material = Tolerator	NA	Not measured	**Response**	Not used	Not used
Plant height	Continuous (cm)	Vertical measurement from the soil surface to the highest green material	Taller plants escape damage by keeping foliage above the flame zone (e.g., Hoffmann et al., 2019)	**Predictor**	Not measured	**Response**	Not used
Stem diameter	Continuous (mm)	The diameter of the main stem at 1 cm above the soil surface	Thicker stems provide more effective insulation against large temperature fluctuations (Midgley et al., 2010).	**Predictor**	Not measured	**Response**	Not used
Main stem length	Continuous (cm)	The length of the main (tallest) stem from the soil surface to the highest bud (excluding the bud itself and any leaves above)	Longer stems have more buds from which to resprout (Meier et al., 2012)	**Predictor**	Not measured	Not used	Not used
Leaves below 1 cm	Continuous (count)	Count of the number of true leaves with petioles emerging from the stem at or below 1 cm	Basal biomass is more sheltered and provides bud banks for resprouting (Clarke et al., 2013; Peìrez‐Harguindeguy et al., 2013)	**Predictor**	Not measured	Not used	Not used
Number of lateral branches	Continuous (count)	Count of the number of lateral branches emanating from the main stem	NA	Not measured	Not measured	**Response**	Not used
Number of stem tips	Continuous (count)	Count of the total number of stem tips of the plant	NA	Not measured	Not measured	Not measured	**Response**
Plant height:diameter ratio	Continuous	The plant height divided by the main stem diameter	NA	Not used	Not measured	**Response**	Not used
Plant height:stem tip ratio	Continuous	The plant height divided by the total number of stem tips	NA	Not measured	Not measured	Not measured	**Response**
Stem regrowth	Continuous (%)	Calculated by the equation (*T/C)100*, where *C* is the average final stem length for control plants and *T* is the average final stem length of treatment plants	NA	Not measured	Not measured	Not measured	**Response**

Note

The format of the trait measurement, the method used to measure the trait, and how the trait was hypothesized to affect seedling survival are indicated. Times at which measurements were taken, that is, immediately prior to the experimental treatment (“Baseline”) with fire or frost, 2 weeks after treatment, 2 months after treatment and/or at the end of the experiment (“Final”) approximately 1 year after seeds were sown, is also provided. Additionally, whether the trait measure was used as predictor or a response variable in analyses is indicated. Baseline measures were often used as covariates in models, as it was expected that initial measures of traits would affect post‐treatment traits. For more details on analyses see the text.

Survival status (dead or alive) was recorded at the end of the experiment to provide information on survival rates for different species under the different treatments. Live plant material present 2 weeks after the treatments was recorded as an indication of which seedlings retained aboveground live foliage after the disturbance (resprouting only started to appear approximately 1 month after treatment). “Live foliage” was defined as any visibly live material such as leaves, stems, and active buds. For seedlings that survived the disturbance, survival strategy was scored. Species that had live material 2 weeks after disturbance, indicating that they were capable of avoiding total aboveground tissue damage, were classified as “disturbance resistors.” In contrast, seedlings that survived solely by resprouting after total aboveground biomass destruction were classified as “disturbance tolerators” (see Archibald et al. (2019) for a discussion of these definitions).

A number of traits were measured prior to disturbance to assess their contribution to seedling survival. These were plant height (height of the highest green material), diameter of the main stem at 1 cm above the soil surface), main stem length, and the number of leaves below 1 cm (Table 1). Taller plants may resist damage by having their foliage and buds above the zone of severe temperatures (Hoffmann et al., 2019). Larger basal diameters could increase survival to disturbance by providing insulation to stem tissues, making the stems more resistant to large temperature fluctuations (Midgley et al., 2010). Plants with longer stems may have higher numbers of epicormic buds, from which new shoots can resprout (Meier, Saunders, & Michler, 2012). Stem length is related to plant height, but compensates for skew‐growing plants. The number of leaves below 1 cm indicates the extent of photosynthetically active resources and buds available to the plant in the basal area, and potentially its ability to survive via resprouting if top killed by a major disturbance event (Clarke et al., 2013; Peìrez‐Harguindeguy et al., 2013).

Plant height, stem length, lateral branching, and stem tip counts were measured postdisturbance to assess changes in architecture due to fire and frost exposure. Main stem length was used to determine the degree of recovery achieved at the end of the experiment. Plant height: stem diameter ratio was taken as an indicator of whether stem growth is long and slender or short and thick. This may indicate whether the seedlings invested more resources into height gain versus lateral growth in response to the treatment; that is, if they are following an “escape” strategy (Dantas & Pausas, 2013; Rozendaal et al., 2015). Also, plant height:stem tip ratio was calculated to determine whether seedlings invested more resources in shorter cagey, or taller growth with fewer branches (Archibald & Bond, 2003).

### 2.3 Analyses

To test for collinearity between predictor variables, Pearson's correlation analysis was conducted between all traits using the control treatment data measured at all time periods. Only control data were used as treatment effects may influence relationships between variables. Seedling age was excluded here as all control seedlings were the same age when measured.

Pearson's Chi‐square test was used to compare seedling survival between treatments (control, fire, or frost) at the end of the experiment. A chi‐squared goodness‐of‐fit test with “fdr” *p*‐value adjustment was performed for pairwise comparisons between treatments.

A generalized linear mixed‐effects model (GLMM) was used to test which traits were significant predictors of seedling survival, with species ID as random effect (Table S3). The predictors used at the outset of this analysis were baseline age (the age of the seedlings on the day they were treated), baseline plant height, baseline number of leaves below 1 cm, baseline stem diameter and treatment (fire or frost—control plants not used). To determine the best subset of predictor variables to use in the GLMM model, a best subsets model selection was used. The combination of factors resulting in the lowest BIC score was then selected for the final GLMM model (Table S3).

For the seedlings that survived treatment, we tested which traits predicted survival strategy (resisting vs. tolerating). A GLMM model was run with treatment (fire or frost, without control plants), baseline age, baseline stem length, baseline stem diameter, baseline number of leaves below 1 cm, as well as the interaction terms between treatment and all traits, included as predictors. Species ID was included as random effect (Table S3). Best subset modeling, using the lowest BIC, was again used to select the best subset of predictors.

To determine the extent of stem regrowth after treatment, the average final stem length was calculated per species for each of the treatments. From this, average percentage stem regrowth was calculated for fire and frost treatment, using the equation $\frac{T}{C}\times 100$, where *C* is the average final stem length for control plants and *T* is the average final stem length of treatment plants. A linear mixed‐effects model (LMM) was fitted to test the effect of treatment on stem length regrowth. Transformed proportion values were used as model input (Table S3).

To test how architectural traits (plant height, stem diameter, number of lateral branches, plant height: stem tip ratio, and final diameter:stem tip ratio) of surviving seedlings were affected by fire and frost, we ran LMM or GLMM models (Table S3). Traits were included as response variables, treatment (fire, frost, or control), and seedling age (at the time of treatment) as predictor variables, and species ID as random effect. Pairwise post hoc comparisons between treatments were done using general linear hypotheses with Bonferroni *p*‐value adjustments to control for falsely rejected hypotheses (the false discovery rate) that could occur with multiple comparisons (Benjamini & Hochberg, 1995).

In addition, to test how architectural traits of resprouted seedlings compared between treatments, LMM or GLMM models were run on a dataset of only the tolerators (i.e., the seedlings that experience top kill but resprouted after disturbance).

Variance inflation factor (VIF) analyses were conducted for each of the above models to test for multicollinearity of predictor variables. VIF measures how much the variance of a regression coefficient is inflated due to multicollinearity in the model (Quinn & Keough, 2002). Variables with a VIF value larger than 5 were removed due to collinearity (James, Witten, Hastie, & Tibshirani, 2013). Because we had different sample sizes across species due to limited germinations or early seedling mortality, all abovementioned analyses were also run excluding the four underrepresented species (fewer than six individuals, Table S2). This did not qualitatively change the conclusions made from the models; therefore, it was decided to include all species in the final analyses.

All analyses were conducted in R version 3.5.2 (R Core Team, 2018) using packages “lme4” version 1.1‐20 (Bates, Mächler, Bolker, & Walker, 2014), “leaps” version 3.0 (Lumley, 2017), “RVAideMemoire” version 0.9‐73 (Hervé, 2019), “multcomp” version 1.4‐8 (Hothorn, Bretz, & Westfall, 2008), and “car” version 3.0‐2 (Fox et al., 2012). Family, link functions, and response variable transformations for all LMM and GLMM models are displayed in Table S3.

## 3 RESULTS

During the fire experiment (Table 2), the calculated fire spread rate was 0.4 m/s and fireline intensity estimated at 1,230.44 kJ s^−1^ m^−1^ based on the equation of Trollope et al. (2002). The lowest night‐time temperature recorded during the frost experiment (Table 2) was −4.71°C at 90 cm above soil level, and −7.85 at tree level (±60 cm above soil surface). Temperatures 10 cm below the soil surface in the soil bags averaged 6.65°C (Table 2).

**TABLE 2:** Summary of conditions during fire and frost treatments

Fire conditions	
Date of treatment	3 May
Time	12:00–13:13
Average relative humidity (%)	46.00
Average air temperature (°C)	25.00
Average wind speed (km/hr)	4.00
Fuel load (kg/ha)	1,708.08
Average fuel moisture (%)	7.77
Total burn time (s)	73.00
Rate of spread (m/s)	0.40
Fire intensity (kJ s^−1^ m^−1^)	1,230.44
Average scorch height (cm)	46.57

Frost conditions	
Date of treatment	16–17 July
Time	17:00 –5:00
Average relative humidity (%)	11.958
Average wind speed (km/hr)	0.00
Air temperature‐90 cm (°C)	Mean: 5.379
	*SE*: 0.086
	Min: −4.705
	Max: 24.102
Air temperature‐60 cm (°C)	Mean: 5.471
	*SE*: 0.070
	Min: −7.847
	Max: 36.703
Soil temperature‐10 cm below soil in soil bags (°C)	Mean: 6.650
	*SE*: 0.051
	Min: −1.097
	Max: 26.057

Note

Rate of spread and fire intensity was calculated from formulas by Trollope et al. (2002).

Pearson's correlation coefficient analysis of the control tree data indicated that there were few significant correlations between the measured traits at −0.7 ≥ *r* ≤ 0.7; (Table S4). All such correlations were between variables representing plant size, namely plant height, stem length, and stem diameter.

Seedling survival was significantly higher in control (100%) than treated (frost: 75%; fire: 66.7%) seedlings (Χ^2^ = 70.35, *df* = 2, *p* < .001). Although the fire‐treated seedlings had lower survival rates than the frost seedlings, this difference was not significant.

Seedling age at the time of treatment, number of leaves below 1 cm, and the main stem diameter before treatment were retained in the best subset model predicting survival. Treatment was removed due to VIF scores above 5 as it was correlated with age (Table 3). This is because the two treatments happened at different times, corresponding to the times at which these disturbances may have happened in the field. The number of leaves below 1 cm and seedling age increased seedling survival (Table 3, Table S3). Diameter was not a significant predictor.

**TABLE 3:** Results for linear mixed‐effects models (LMM) or generalized linear mixed‐effects models (GLMM) (after selecting for the best subset of predictors) testing for the best predictors of seedling survival, survival strategy (resistance vs. tolerance) and stem regrowth

Fixed effects	Estimate	Std. error	*z*‐value	*p*‐value	VIF
Survival (GLMM)					
Intercept	−1.592	0.916	−1.739	NS	‐
Age	0.019	0.007	2.690	**	1.455
Leaves below 1 cm	0.308	0.116	2.649	**	1.049
Diameter	−0.082	0.074	−1.104	NS	1.419

Random effects	Variance	Std. dev.
Survival (GLMM)		
Species ID	1.486	1.219

Fixed effects	Estimate	Std. error	*z*‐value	*p*‐value	VIF
Survival strategy (GLMM)					
Intercept	−3.484	0.808	−4.314	***	‐
Treatment	1.918	0.497	3.859	***	1.331
Stem length	0.038	0.019	2.030	*	1.055
Leaves below 1 cm	0.765	0.180	4.261	***	2.044
Treatment × Leaves below 1 cm	−0.779	0.233	−3.347	***	1.935

Random effects	Variance	Std. dev.
Survival strategy (GLMM)		
Species ID	3.635	1.907

Fixed effects	Estimate	Std. error	*t*‐value	*p*‐value	VIF
Stem regrowth (LMM)					
Intercept	−0.512	0.256	−2.000	NS	‐
Treatment	−0.023	0.269	−0.085	NS	‐

Random effects	Variance	Std. dev.
Stem regrowth (LMM)		
Species ID	0.498	0.706
Residual	0.486	0.697

Note

Variance inflation factors (VIF) indicates multicollinearity of predictor variables. R^2^ and model *p*‐values are given in Table S3. Treatment = Fire or Frost; Age = seedling age at which treatment was applied; Number of leaves = the number of leaves below 1 cm before treatment, Diameter = main stem diameter at 1 cm before treatment. Stem length = main stem length before treatment. NS: not significant.

* *p* < .05;

** *p* < .01;

*** *p* < .0001

**FIGURE 2:**
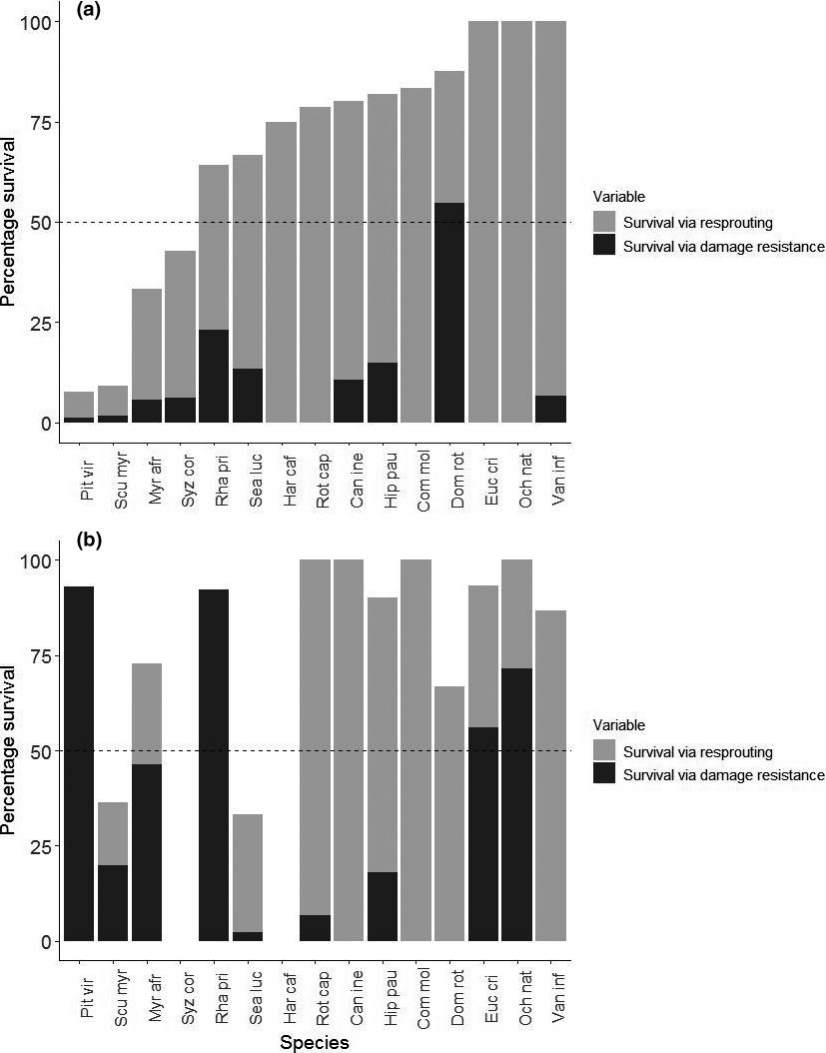
Stacked plot of tree seedling survival strategies. Bars illustrate percentage seedling survival and percentage individuals with live foliage remaining 2 weeks after treatment for tree seedlings treated by (a) fire and (b) frost. Species represented by fewer than six individuals were not included in this figure. Species were considered “resistors” if the percentage individuals with live foliage directly after treatment exceeded the 50% threshold (dashed line). Species were classified as “tolerators” if the percentage resprouted plants exceeded the 50% threshold. Can ine = *Canthium inerme;* Com mol = *Combretum molle;* Euc cri = *Euclea crispa;* Dom rot = *Dombeya rotundifolia;* Har caf = *Harpephyllum caffrum;* Hip pau = *Hippobromus pauciflorus*; Myr afr = *Myrsine africana*; Och nat = *Ochna natalitia;* Pit vir = *Pittosporum viridiflorum;* Rha pri = *Rhamnus prinoides;* Rot cap = *Rothmannia capensis*; Scu myr = *Scutia myrtina;* Sea luc = *Searsia lucida*; Syz cor = *Syzygium cordatum;* Van inf = *Vangueria infausta*

Many species displayed different survival responses to fire than to frost treatments (Figure 2). Some species that survived frost well were adversely affected by fire and *vice versa*. Most species could be classified as fire tolerators (i.e., >50% of survivors resprouted). Only one species (*Dombeya rotundifolia*) could be considered a fire resistor (>50% of individuals retaining live foliage after fire). In contrast, four species could be classified as frost resistors (Figure 2). Interestingly, *D. rotundifolia* was not among the frost resistors. Seven species displayed a frost tolerator response. Treatment, stem length, and the number of leaves below 1 cm were significant predictors of survival strategy (Table 3). Increased stem length was associated with greater resisting capability to both treatments. We also found a significant interaction effect between treatment and leaves below 1 cm. More leaves below 1 cm resulted in greater resistance against fire damage, but not against frost. No other significant interaction effects were found between treatment and any of the other predictor variables used in the model (Table S3).

**FIGURE 3:**
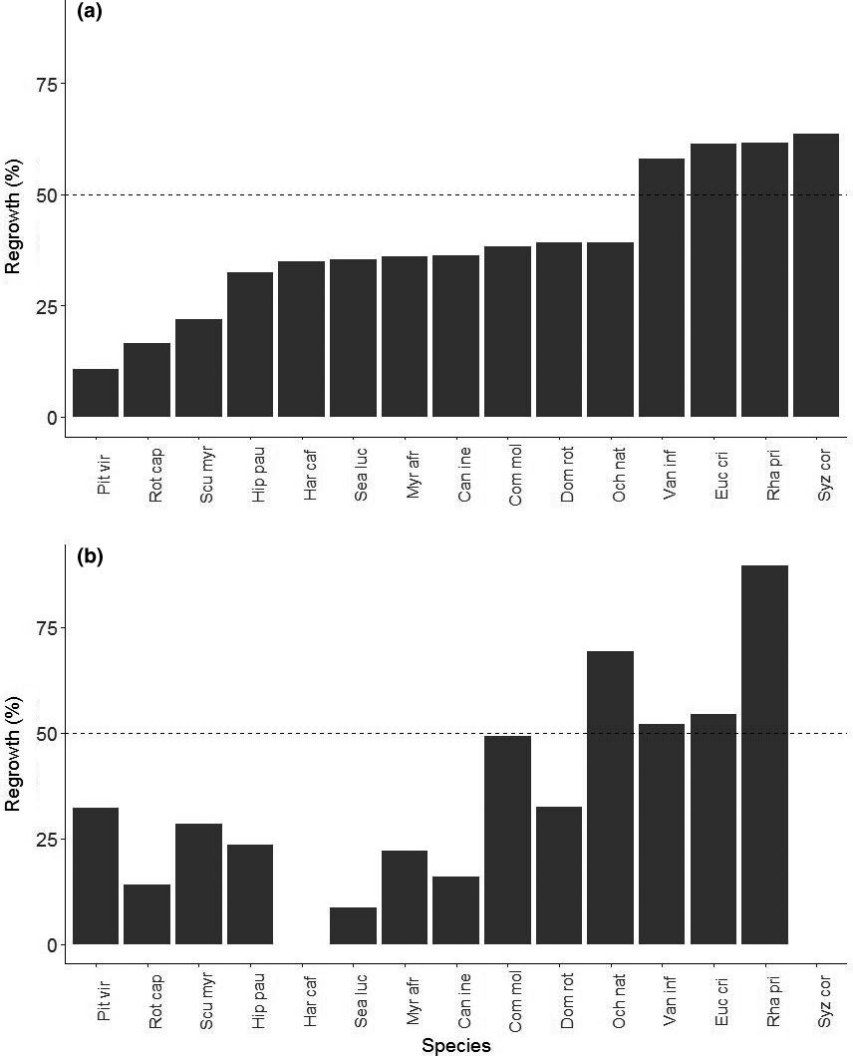
Percentage stem length regrowth for tree seedlings as a result of fire (a) and frost (b) damage. Species represented by fewer than six individuals were not included. Dashed line represents 50% cut‐off for stem regrowth. Can ine = *Canthium inerme;* Com mol = *Combretum molle;* Euc cri = *Euclea crispa;* Dom rot = *Dombeya rotundifolia;* Har caf = *Harpephyllum caffrum;* Hip pau = *Hippobromus pauciflorus*; Myr afr = *Myrsine africana*; Och nat = *Ochna natalitia;* Pit vir = *Pittosporum viridiflorum;* Rha pri = *Rhamnus prinoides;* Rot cap = *Rothmannia capensis*; Scu myr = *Scutia myrtina;* Sea luc = *Searsia lucida*; Syz cor = *Syzygium cordatum;* Van inf = *Vangueria infausta*

**FIGURE 4:**
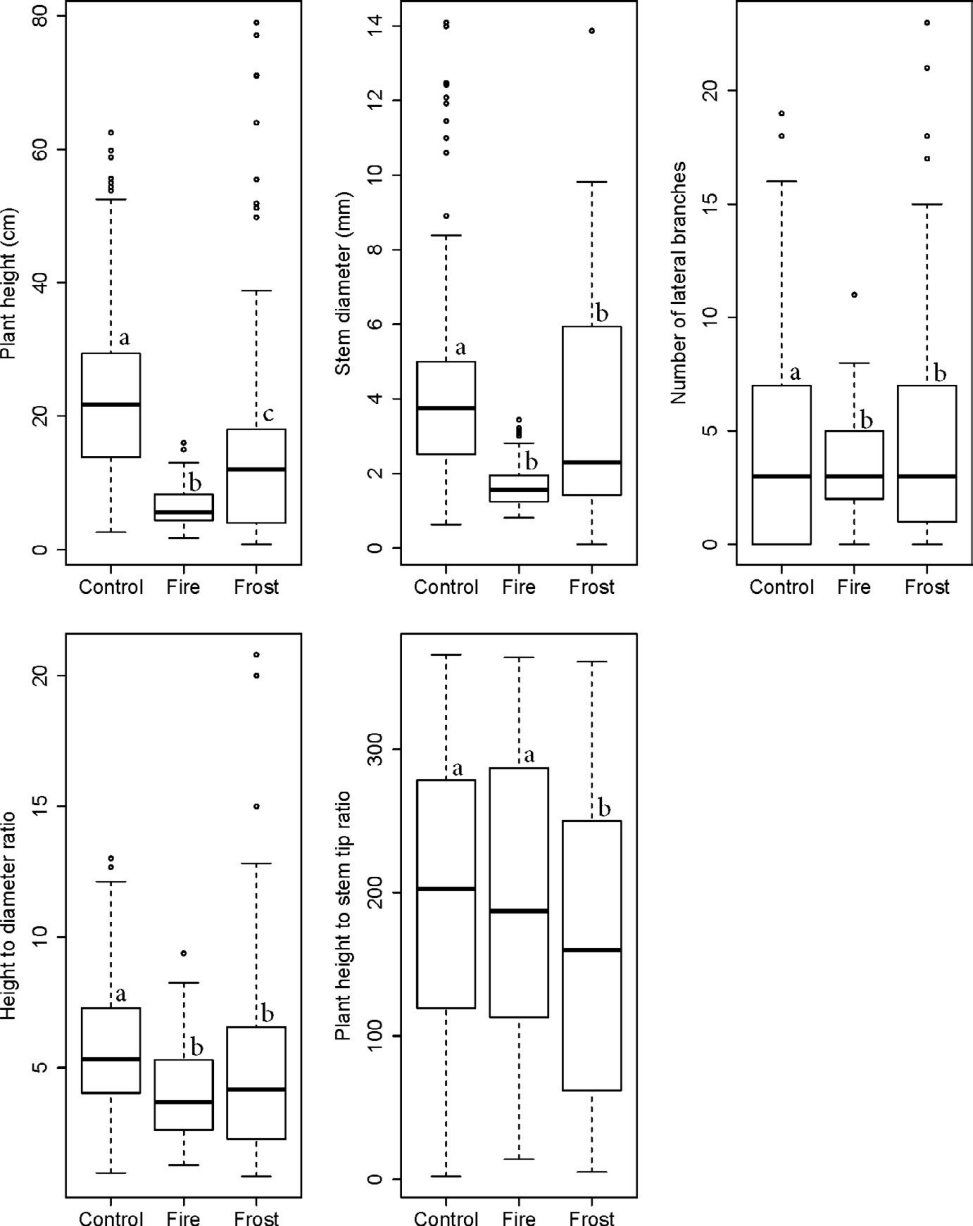
Architectural trait differences of surviving tree seedlings between three treatment groups (C = control, FI = fire, and FR = frost). All response variables were measured 2 months after treatment, except diameter:stem tip ratio which was measured at the end of the experiment. Different letters above plots indicate significant differences in means between treatments at *p* ≤ .05

There was variation in the degree to which species recovered lost biomass after disturbance, but regrowth rates for any particular species after fire versus frost were similar (Table 3, Figure 3). As for architectural trait differences of surviving seedlings 2 months post‐treatment, control seedlings were taller, with larger stem diameters and more lateral branches than treated seedlings (Tables S5 and S6; Figure 4). After 2 months of recovery, seedlings that were burned were shorter than seedlings that received frost, though stem diameter did not differ between treatments. At the end of the experiment, burned seedlings had significantly higher plant height:stem tip ratios than frost‐treated seedlings, indicating that they had a lower degree of branching in relation to height, compared to seedlings exposed to frost. However, the models containing only resprout data with the same equations indicated that there were no significant differences for any of the traits between the fire and frost treatment when only considering the individuals that resprouted (Table S7).

## 4 DISCUSSION

### 4.1 Seedling survival

Survival rates in response to fire and frost were similar under both treatments. Therefore, we cannot conclude whether fire or frost is more important in preventing the establishment of tree seedlings in these systems. To our knowledge, this study represents one of the first that explicitly contrasts the effects of fire and frost on young tree seedlings across a range of species. While the role of fire and frost in limiting woody cover is often acknowledged, few studies specifically contrast their respective effects without them being confounded by other factors such as herbivory, rainfall, and soil moisture (Hirota, Holmgren, Van Nes, & Scheffer, 2011; Murphy & Bowman, 2012). For example, Holdo (2007) demonstrated in a simulation model that fire and herbivory by elephants were particularly important drivers of savanna change, and that frost alone was less effective in causing change in woody cover. On the other hand, in a review about the drivers of woody cover, fire (but also grass competition and herbivory) were posited to be particularly important in maintaining grasslands globally, the effects of frost were not specifically interrogated (Bond, 2008). However, there is evidence for frost as the main driver of woody cover in some systems (Brando & Durigan, 2005; Hoffmann et al., 2019; Joshi, Ratnam, & Sankaran, 2019).

Overall, seedlings were remarkably resilient to fire and frost damage. Our results suggest that a wide variety of indigenous tree species are potentially capable of surviving fire and frost damage during their first growth year. More than half of the seedlings considered in this study survived the treatments. Overall, 67% and 75% of seedlings survived fire and frost, respectively, and only four species showed less than 50% survival after a single fire or frost event (Figure 2; Table S2). Other studies have found mixed results: One study found that young seedlings of four African savanna tree species could survive three consecutive cooling events to −5°C in a cold chamber (O'Keefe, Nippert, & Swemmer, 2016), while another found relatively low survival rates for tree seedlings subjected to their first fire event in field observations (Gignoux et al., 2009). Differences between our observations and other studies could be due to the experimental nature of our study: seedlings were well‐watered, grown in nutrient‐rich soils and grew without grass or other competitors; therefore, seedlings had obtained fairly large sizes at the time of experimental treatment. Additionally, fire intensity of our burn was low (Trollope et al., 2002), with average leaf scorch heights of less than one meter recorded directly after the burn. Also, frost was applied as a single event rather than repeated throughout the dry cold season. Therefore, natural fire regimes and frost events may result in different mortality rates.

### 4.2 Plant traits that aid seedling survival

The ability of woody seedlings to survive disturbance will be influenced by certain plant traits. Seedling survival increased with increased number of leaves in the basal region prior to treatment. Fire and frost are mostly top‐kill events (especially for seedlings smaller than 4 m) with less damage to foliage close to the soil surface than to aerial foliage (Finckh et al., 2016; Whitecross et al., 2012); therefore, photosynthetic sources and extensive bud banks in the basal region increase tolerance to these disturbances (Clarke et al., 2013). Basal resprouting seems to be a prominent fire tolerance strategy, even for mature woody species, in African savannas compared with other fire‐prone systems (Clarke et al., 2013), and may also serve as an adaptation to frost. This may be a particularly important character in young seedlings, as they have not had the time to build up some of the defenses (e.g., height or stem diameter) that adult trees possess.

Our findings that older seedlings had higher survival rates agree with other studies assessing fire (Gignoux et al., 2009; Hoffmann, 2000; Radford, Nicholas, & Brown, 2001) and frost (Gusta & Wisniewski, 2013; Molina, Hadad, Domínguez, & Roig, 2016) survival. Older individuals would have had more time to accumulate above‐ and belowground resources, providing capacity to either resist damage to stems or regrow from stored reserves after top kill (Gignoux et al., 2009).

We found that neither seedling height nor stem diameter was significant predictors of survival. Other studies have shown that the critical escape height for fire and frost ranges between 2–4 m in African savannas (Trollope et al., 2002; Wakeling et al., 2012; Whitecross et al., 2012). The mean height of our seedlings at time of treatment was 22.24 cm (std. dev: 17.39). The stem diameter above which stem survival after exposure to fire increases varies between species (Gignoux et al., 1997; Hoffmann, Orthen, & Do Nascimento, 2003). Diameters between 20–40 mm have been identified as thresholds for 100% survival of some species (Gignoux et al., 1997; Hoffmann et al., 2003), and diameters between 4.5 and 10.32 cm for 50% survival (Hoffmann et al., 2003); though these values will also vary with fire intensity. The mean diameter of our seedlings at time of treatment was 3.95 mm (std. dev: 3.12). Therefore, even though our seedlings were probably considerably larger than they would have been at the end of their first season under natural conditions due to the conducive experimental conditions under which they were grown, they still did not achieve sufficient height or thick enough stems to be completely resistant to fire and frost after one growing season.

Our results support the notion that the traits promoting survival in seedlings are fundamentally different from those of older saplings or mature trees. It is known that mature trees that are taller (Balfour & Midgley, 2006; Bond, 2008; Burrows et al., 2008; Higgins et al., 2000) with thicker stems (Michaletz et al., 2012; Midgley et al., 2011) and other traits not considered here such as thick bark and bud protection structures (Gignoux et al., 1997; Hoffmann et al., 2009; Midgley et al., 2010; Waters et al., 2010) have increased survival under fire and frost damage.

### 4.3 Seedling survival strategies

The traits that promote survival in response to disturbance may be subdivided into those that are helpful for damage resistance and those that aid damage tolerance (resprouting). Our results support the idea that different plant functional traits are associated with resisting and tolerating fire and frost. More basal leaves and longer stems aided resistance (Table 3, Table S3). None of the traits considered here were specifically associated with seedling tolerance. Other traits that were not measured, such as those associated with belowground resource allocation and basal bud morphology, may be specifically important for tolerating top kill (Charles‐Dominique, Beckett, Midgley, & Bond, 2015; Gignoux et al., 2009). Resprouters may benefit from having large belowground bud banks, belowground storage, and well‐protected buds, which may be traded off against vertical growth to persist in damage‐prone environments until a way is found to grow past the critical vulnerable height (Gignoux et al., 2009).

Some traits that prevent or limit damage from extreme heat spikes, as would be experienced during fires, may simultaneously serve as protection against extreme cold (Gurskaya & Shiyatov, 2006; Molina et al., 2016; Treter & Block, 2004). Childes and Walker (1987) found that mature fire‐resistant African tree species also tend to be frost hardy. It is often difficult to establish direct causal relationships between the evolution of traits and their current function. For example, it has been suggested that traits that aid survival under frost may initially have evolved as an adaptation to fire (Lamont, He, & Pausas, 2017). On the other hand, it has often been suggested that fire adaptations originally evolved in response to drought (Axelrod, 1980; Blödner, Skroppa, Johnsen, & Polle, 2005; López‐Soria & Castell, 1992).

However, our results illustrate that not all traits may be equally beneficial for resisting both disturbances. We found discrepancies in the species' abilities to resist fire and frost, and that none of the species could resist both treatments equally well. Seedlings with larger numbers of leaves in the basal region were better resistors to fire, while this trait was less helpful to resist frost. Tree seedlings are therefore able to resist fire by keeping their foliage low to the soil surface. In contrast, none of the traits we measured was specifically helpful for resisting frost. Other factors not examined here could affect resistance to frost. For example, caginess (i.e., a higher degree of branching) might provide a higher density of leaves, which could provide cold insulation to the main stem. Temperatures within canopies, at least for mature trees, can be significantly warmer inside than outside the canopies during frost (Whitecross et al., 2012), and frost‐hardy species have been shown to possess shorter, more branched stems compared with frost‐sensitive species (Palta & Li, 1979). In our study, frost survivors were overall more cagey compared with fire‐treated trees after the treatment (discussed in more detail below), although this caginess was not a reaction of resprouters to frost, but rather the result of less top kill. This change in architecture might, however, provide additional protection to a successive frost event.

Other adaptations not considered may be unique for either frost or fire resistance since there are differences in the way fire and frost cause physiological damage. Fire‐adaptation traits reduce stem flammability and protect the cambium, vascular tissues, and buds from heat damage (Balfour & Midgley, 2006; Gignoux et al., 1997, 2009; Hoffmann & Solbrig, 2003). From temperate zones which experience longer (several‐hour) frost events with low diurnal temperature variation, adaptations involve preventing intracellular freezing and tolerating extracellular ice crystal formation in plant tissues (Gusta & Wisniewski, 2013). (Whether such frost adaptations have also evolved in areas with short frost events with large diurnal temperature variations, as found in subtropical savannas, has not been investigated). Apart from traits already mentioned such as thick bark (Schafer, Breslow, Hohmann, & Hoffmann, 2015), well‐protected buds (Charles‐Dominique et al., 2015), and belowground storage organs (Maurin et al., 2014), trees with smaller leaf area possess lower time to ignition, burn duration, and mean mass loss rate (Krix & Murray, 2018), which may decrease fire damage. Frost‐hardy plants tend to have smaller, thicker leaves (Palta & Li, 1979) with decreased leaf wettability (through waxy cuticles or pubescence) (Aryal & Neuner, 2010). The ability of some frost‐hardy woody species to retain liquid cellular water under subzero conditions (supercooling) by preventing ice nucleation has also been illustrated (Wisniewski, Bassett, & Gusta, 2003). Hydraulic system failure resulting from cavitation after a freeze‐thaw event occurs because air bubbles remain in the xylem sap after degassing of the frozen xylem, and this process therefore differs from cavitation caused by tension in the vessels during extreme drought or heat during a fire (Dickison, 2000). Small vessel diameter is particularly important in preventing cavitation due to freeze‐thaw events since larger tracheary elements form larger air bubbles during subzero conditions (Dickison, 2000). Evidence that fire mortality also occurs through cavitation means that small vessel diameters (and particularly hydraulic segmentation) may also contribute to fire resistance (West et al., 2016).

### 4.4 Changes in seedling architecture after fire and frost damage

Tree architecture can take on a very different trajectory after damage compared with undamaged trees (Archibald & Bond, 2003; Holdo, 2006; Klimes, 2007). Although the recovery of stem length after fire and frost was similar under both treatments, which coincides with other studies (Brando & Durigan, 2005), interspecific differences existed, where some species recovered a larger percentage of their stem length after damage than others. Almost all of the species that could resist damage to their aboveground biomass under fire and frost still suffered more than 50% loss of their stem length after treatment. The plant height and stem diameter of seedlings after 2 months of recovery were also significantly reduced in seedlings damaged by both treatments. The buildup of height and stem thickness “debt” may have implications for seedling survival during subsequent disturbance events: Smaller seedlings with thinner stems are more vulnerable to large thermal fluctuations and may suffer increased mortality (Gignoux, Konaté, Lahoreau, Le Roux, & Simioni, 2016; Lawes, Adie, Russell‐Smith, Murphy, & Midgley, 2011; Midgley et al., 2010). Seedlings face trade‐offs between investing in stem length and resisting damage, and building belowground storage or basal defense mechanisms (e.g., bark thickness and bud protection) to improve resprouting ability (Gignoux et al., 2009). Spending time investing in belowground resources for instance may limit the seedling's ability to gain height.

The architecture of frost‐treated seedlings differed from those that received fire in that they had a higher degree of branching in relation to height. Since fire destroyed more biomass than frost, seedlings mostly created new stems from the base after fire, whereas they were more often able to retain their original branches after frost damage. These differences therefore appear to be due to the fact that more fire‐affected trees resprouted, rather than intrinsic differences in response to each type of damage when the models were run with only the individuals that resprouted.

### 4.5 Implications for grassland structure and management

While seedling survival was high under fire and frost, the severe stunting effects of these treatments (especially fire) suggests that, once established, trees in these open grassland systems may be subjected to a seedling release bottleneck (i.e., seedlings can survive but not grow to adulthood). This is consistent with the findings of others who found that fire mainly suppresses seedling growth and has less effect on seedling mortality (Staver, Bond, Stock, Van Rensburg, & Waldram, 2009). However, the question remains whether seedlings are able to recruit in these grasslands (Russell, Tedder, & Demmer, 2019). Competition for root space may be severe during the germination phase, especially considering the fact that most grass roots are concentrated within the first 10 cm of soil (Wakeling, Bond, Ghaui, & February, 2015). It is therefore likely that the lack of root gaps may have contributed significantly to the relatively open structure of this system.

High‐intensity fires are more sufficient in maintaining open grassy areas, as they kill more woody seedlings and cause more damage to aboveground biomass (Morrison & Renwick, 2000; Trollope et al., 2002). While fire rarely burns more than once a year due to fuel limitations, the cumulative effects of repeated frost events during a single season may further reduce survival probability (Joshi et al., 2019). Also, the combined effect of fire and frost may be more effective in killing seedlings than either disturbance alone (Holdo, 2007). In areas where frosts have become less frequent, as has been observed in Africa and beyond (Midgley & Lötze, 2008; Rigby & Porporato, 2008), fire alone may not be able to prevent recruitment, and an increase in woody cover may be expected.

The woody species composition of an open grassland system will almost certainly be influenced by phenomena that affect the timing and frequency of seedling exposure to damage, as mortality was found to be dependent on seedling age. Also, the vulnerability of seedlings to disturbances may change with the season. Seedlings that receive fire in autumn may still have fresh, humid foliage, which may make them less flammable compared to trees with dry foliage in late winter (Hoffmann et al., 2019). For deciduous species, the effect of disturbances may be less severe after they have shed their leaves (Blumler, 1991) (Our study area has few fully deciduous species (Table S1), and of the deciduous species we planted, only *Pterocarpus angolensis* became fully deciduous fairly late in the season, which may be due to the mild conditions in the greenhouse). Therefore, changes in fire regime and climates (esp. changes that bring about less frost and reduced fire intensity and frequency) are likely to result in changes in the composition and the structure of the woody components of these systems (Enright, Fontaine, Bowman, Bradstock, & Williams, 2015).

## CONFLICT OF INTEREST

Hereby, it is confirmed that none of the authors have any conflict of interest concerning this work.

## AUTHOR CONTRIBUTIONS

**Monique Botha:** Data curation (lead); formal analysis (lead); investigation (lead); project administration (lead); writing – original draft (lead). **Sally Archibald:** Conceptualization (equal); formal analysis (supporting); funding acquisition (supporting); methodology (equal); supervision (lead); visualization (equal); writing – review & editing (equal). **Michelle Greve:** Conceptualization (lead); formal analysis (supporting); funding acquisition (lead); methodology (lead); project administration (supporting); supervision (supporting); visualization (equal); writing – review & editing (equal).

## Supporting information

Supplementary Material

## Data Availability

Data have been submitted to Dryad for review: https://datadryad.org/stash/share/7KE7jOUKVnvj6GZwAP36usJXB9tIOc5G7PXGpg8SwFA.
