# Supplementary material for: What drives grassland‐forest boundaries? Assessing fire and frost effects on tree seedling survival and architecture
Source: Ecol Evol. 2020 Sep 20;10(19):10719–34. doi: 10.1002/ece3.6730 (PMC7548188; doi:10.1002/ece3.6730)
Supplement: Supplementary file 1 — Supplementary Material [file ECE3-10-10719-s001.docx]

**Table S1:** Table of the species and their respective families on which the effects of fire and frost were measured. Additionally, the habitat description, growth-form, branching architecture and leaf phenology of each species are provided. Information for the latter four columns were obtained from Palgrave (2015), Schmidt et al. (2002) and Van Wyk & Van Wyk (2013).

| **Family** | **Species** | | **Habitat description** | **Growth-form description** | **Typical branching architecture** | **Leaf phenology** |
| --- | --- | --- | --- | --- | --- | --- |
| Rubiaceae | *Canthium inerme* Kuntze | | Coastal and montane forest, in woodland, along streams and high-altitude grasslands among rocks | Small to medium-sized tree | Single stem, branching low down | Evergreen |
| Combretaceae | *Combretum kraussii* Hochst. | | Evergreen forest and forest margins | Medium to tall densely leafy tree | Single-stemmed (to 0.8 m diameter) or multi-stemmed, branching low down | Deciduous or semi-deciduous |
| Combretaceae | *Combretum molle* R.Br. ex G.Don | | Open woodland and bushveld | Small to medium-sized tree | Single upright stem | Deciduous or semi-deciduous |
| Malvaceae | *Dombeya rotundifolia* Hochst. | | Bushveld and woodlands, often with termite mounds | Small erect tree | Single upright stem | Deciduous |
| Sapotaceae | *Englerophytum magalismontanum* (Sond.) T.D.Penn. | | Rocky outcrops, also occurring in forest, wooded ravines and along river banks | Small to medium-sized tree | Single short, stout, twisted stem | Evergreen |
| Ebenaceae | *Euclea crispa* (Thunb.) Gürke | | In bush clumps in open grasslands, open woodland and bushveld, often among rocks, at forest margins | Shrub or small tree | Multi- or single-stemmed, stems crooked, densely branching low down | Evergreen |
| Anacardiaceae | *Harpephyllum caffrum* Bernh. | | Occurring in forest | Medium to large tree | Single clean, straight stem | Evergreen |
| Sapindaceae | *Hippobromus pauciflorus* Radlk. | | Riverine thicket and scrub, along stream banks and margins of evergreen forest | Shrub or small, densely leafy tree | Stem single, branching low down | Semi-deciduous |
| Myrsinaceae | *Myrsine africana* L. | | Wide range of altitudes, among rocks, in open woodland and margins of evergreen forests, often a pioneer species | Shrub or small tree | Multi- or single slender stem | Evergreen |
| Ochnaceae | *Ochna natalitia* Walp. | | Bushveld and grassland, in understorey of forested kloofs and margins of evergreen forests | Shrublet, shrub or small tree | Single-stemmed, occasionally rhizomatous | Evergreen |
| Pittosporaceae | *Pittosporum viridiflorum* Sims | | Wide range of altitudes, in deciduous woodlands, scrub, riverine thicket and evergreen forest, also on rocky outcrops | Small to large tree | Single-stemmed | Deciduous or evergreen |
| Fabaceae | *Pterocarpus angolensis* DC. | | Grassland and open bushveld | Medium to large tree with wide spreading crown | Single-stemmed | Deciduous |
| Rhamnaceae | *Rhamnus prinoides* L'Hér. | | Widespread and locally common at medium to high altitudes, along watercourses, in riverine forest and margins of evergreen forest | Scrambling shrub or small tree | Stem single, branching low down | Evergreen |
| Rubiaceae | *Rothmannia capensis* Thunb | | Wide range of altitudes (sea level to 1600m) in evergreen forest, wooded ravines, and on rocky hillsides | Small to medium-sized tree, occasionally large (to 15 m) in tall forest | Single upright stem | Evergreen |
| Rhamnaceae | *Scutia myrtina* (Burm.f.) Kurz | | Coastal and scrub forest and margins of evergreen forest | Liane, climbing shrub or small to medium-sized tree | Usually multi-stemmed or single stem branching low down | Evergreen |
| Anacardiaceae | *Searsia lucida* (L.) F.A.Barkley | | Occurring in scrub and forest, from sea level to 2000m | Shrub or small tree | Single stem, highly branched low down | Evergreen |
| Table S1 cont. |  | |  |  |  |  |
| Myrtaceae | *Syzygium cordatum* Hochst.ex C.Krauss. | | Almost always occurring near water, in riverine thicket and forest, sometimes forming stands in swamp thicket | Medium to large tree | Single upright stem | Evergreen |
| Rubiaceae | *Vangueria infausta* Burch. | | Occurring in bushveld, open woodland, among rocks and in coastal scrub | Small, stout tree | Usually single stemmed, branching low down | Deciduous or semi-deciduous |
| Rhamnaceae | *Ziziphus mucronata* Willd. | | Wide variety of habitats, in open woodland, often in alluvial soils along rivers, frequently on termite mounds | Shrub or small to medium-sized tree | Usually single stemmed | Deciduous or semi-deciduous |
|  | |  |  |  |  |  |

**Table S2:** Summary of total number of individual tree seedlings subjected to fire and frost, number of living and dead individuals at the end of the experiment, number of individuals with live foliage remaining 2 weeks after treatment and the number of individuals that resprouted after total destruction of aboveground biomass after treatment by fire and frost. The number of control seedlings by species, used for measurements of functional traits, are also shown.

|  | **Fire** | | | | | **Frost** | | | | | **Control** | |
| --- | --- | --- | --- | --- | --- | --- | --- | --- | --- | --- | --- | --- |
|  | Total  individuals | Surviving  green material | Resprouts | Alive | Dead | Total  individuals | Surviving  green material | Resprouts | Alive | Dead | Total individuals |  |
| *Canthium inerme* | 15 | 2 | 10 | 12 | 3 | 15 | 0 | 15 | 15 | 0 | 15 |  |
| *Combretum krausii* | 2 | 0 | 0 | 0 | 2 | 2 | 0 | 2 | 2 | 0 | 3 |  |
| *Combretum molle* | 6 | 0 | 5 | 5 | 1 | 9 | 0 | 9 | 9 | 0 | 8 |  |
| *Dombeya rotundifolia* | 8 | 5 | 2 | 7 | 1 | 9 | 0 | 6 | 6 | 3 | 9 |  |
| *Englerophytum magalismontanum* | 2 | 0 | 1 | 1 | 1 | 1 | 1 | 0 | 1 | 0 | 2 |  |
| *Euclea crispa* | 14 | 0 | 14 | 14 | 0 | 15 | 9 | 5 | 14 | 1 | 15 |  |
| *Harpephyllum caffrum* | 8 | 0 | 6 | 6 | 2 | 8 | 0 | 0 | 0 | 8 | 8 |  |
| *Hippobromus pauciflorus* | 11 | 2 | 7 | 9 | 2 | 10 | 2 | 7 | 9 | 1 | 9 |  |
| *Myrsine africana* | 12 | 2 | 2 | 4 | 8 | 11 | 7 | 1 | 8 | 3 | 11 |  |
| *Ochna natalitia* | 7 | 0 | 7 | 7 | 0 | 7 | 5 | 2 | 7 | 0 | 8 |  |
| *Pittosporum viridiflorum* | 13 | 1 | 0 | 1 | 12 | 14 | 13 | 0 | 13 | 1 | 15 |  |
| *Pterocarpus angolensis* | 1 | 0 | 1 | 1 | 0 | 1 | 0 | 0 | 0 | 1 | 1 |  |
| *Rhamnus prinoides* | 14 | 5 | 4 | 9 | 5 | 13 | 12 | 0 | 12 | 1 | 10 |  |
| *Rothmannia capensis* | 14 | 0 | 11 | 11 | 3 | 15 | 1 | 14 | 15 | 0 | 15 |  |
| *Scutia myrtina* | 11 | 1 | 0 | 1 | 10 | 11 | 4 | 0 | 4 | 7 | 11 |  |
| *Searsia lucida* | 15 | 3 | 7 | 10 | 5 | 15 | 1 | 4 | 5 | 10 | 15 |  |
| *Syzygium cordatum* | 7 | 1 | 2 | 3 | 4 | 7 | 0 | 0 | 0 | 7 | 7 |  |
| *Vangueria infausta* | 15 | 1 | 14 | 15 | 0 | 15 | 0 | 13 | 13 | 2 | 15 |  |
| *Ziziphus mucronata* | 2 | 0 | 2 | 2 | 0 | 2 | 2 | 0 | 2 | 0 | 2 |  |

**Table S3:** Summary of models run. Model equations represent best subset models after selection by BIC. Family and link function specifications, data transformations, list of predictors dropped by best subset selection (NA indicates no best subset model was used), model R^2^ values associated with fixed effects (marginal) and random effects (conditional), and model significance for linear mixed-effects models (LMM) or generalized linear mixed-effects models (GLMM) are shown. (1|Species) indicates random effect. NS: not significant; *: p≤0.05; **: p<0.001; ***: p<0.0001.

| **Data used** | | **Model** | | **Model equation** | | | **Family** | **Link function** | **Response variable transformations** | **Predictors dropped by BSS model** | **Marginal R^2^** | **Conditional R^2^** | **Model p- value** | |
| --- | --- | --- | --- | --- | --- | --- | --- | --- | --- | --- | --- | --- | --- | --- |
| All (Fire, Frost) | | Species survival (GLMM) | | SurvivalF ~ Age0 + Leaves0 + Diam0 + (1\|Species) | | | binomial | logit | none | Hgreenmat0 | 0.080 | 0.386 | *** | |
| Survivors (Fire, Frost) | | Survival strategy (GLMM) | | Strategy ~ StemL0 + Leaves0 + Treatm × Leaves0 + (1\|Species.name) | | | binomial | logit | none | Diam0;  Treatm × Diam0;  Treatm × Age0;  Treatm × StemL0 | 0.201 | 0.620 | *** | |
| Survivors (Control, Fire, Frost) | | Stem regrowth (LMM) | | Regrowth~Treatm + (1\|Species) | | | NA | NA | logit (on fraction data) | NA | 0.0001 | 0.507 | NS | |
| Survivors (Control, Fire, Frost) | | Plant height (LMM) | | Height2 ~ Treatm + Height0 + Age0 + (1\|Species) | | | NA | NA | square-root | NA | 0.363 | 0.674 | *** | |
| Survivors (Control, Fire, Frost) | | Stem diameter (LMM) | | Diam2 ~ Treatm + Diam0 + Age0 + (1\|Species) | | | NA | NA | log | NA | 0.239 | 0.602 | *** | |
| Survivors (Control, Fire, Frost) | | Lateral branches (GLMM) | | Latbranch2 ~ Treatm + StemL0 + Age0 + (1\|Species) | | | Poisson | log | none | NA | 0.049 | 0.772 | *** | |
| Survivors (Control, Fire, Frost) | | Plant height:diameter ratio (LMM) | | Height:Diam2~ Treatm + Age0 + (1\|Species) | | | NA | NA | log | NA | 0.182 | 0.493 | *** | |
| Survivors (Control, Fire, Frost) | | Plant height:stem tip ratio (LMM) | | Height:stemtip ~ Treatm + AgeF + (1\|Species) | | | NA | NA | square-root | NA | 0.013 | 0.536 | * | |
| Resprouts (Fire, Frost) | | Plant height (LMM) | | Height2 ~ Treatm + Height0 + Age0 + (1\|Species) | | | NA | NA | log | NA | 0.289 | 0.526 | *** | |
| Resprouts (Fire, Frost) | | Stem diameter (LMM) | | Diam2 ~ Treatm + Diam0 + (1\|Species) | | | NA | NA | square-root | NA | 0.071 | 0.827 | *** | |
| Resprouts (Fire, Frost) | | Lateral branches (GLMM) | | Latbranch2 ~ Treatm + StemL0 + Age0 + (1\|Species) | | | Poisson | log | none | NA | 0.137 | 0.539 | *** | |
| Resprouts (Fire, Frost) | | Plant height:diameter ratio (LMM) | | Height:Diam2~ Treatm + Age0 + (1\|Species) | | | NA | NA | log | NA | 0.069 | 0.533 | ** | |
| Resprouts (Fire, Frost) | | Plant height:stem tip ratio (LMM) | | Height:stemtip ~ Treatm + AgeF + (1\|Species) | | | NA | NA | log | NA | 0.017 | 0.330 | NS | |
| **SurvivalF =** Species survival status at end of experiment; **Age0 =** baseline seedling age at time of treatment; **Leaves0** = baseline number of leaves below 1 cm; **Diam0** = baseline stem diameter;  **Species =** Species name; **Strategy =** Resistor/Tolerator; **StemL0** = baseline stem length; **Regrowth** = stem recovery at end of experiment; **Treatm =** Treatment; **Height2** = plant height two months after treatment; **Height0** = Baseline plant height; **Diam2** = stem diameter two months after treatment; **Latbranch2** = number of lateral branches two months after treatment; **Height:Diam2** = height to diameter ratio two months after treatment; **Height:stemtip** = height to stem tip ratio; **AgeF** = seedling age at end of experiment | | | | | | | | | | | | | |  |
|  |  | |  | |  |  | |  | | | | | |  |

**Table S4:** Correlation matrix showing Pearson correlation coefficients between trait values. Shaded cells indicate correlations at r ≥ |0.7|.

|  | **Height0** | **StemL0** | **Diam0** | **Leaves0** | **Height:Diam0** | **Height2** | **Diam2** | **Latbranch2** | **AgeF** | **HeightF** | **StemLF** | **DiamF** | **LeavesF** | **Stemtips** | **Height:stemtip** | **Height:Diam2** |
| --- | --- | --- | --- | --- | --- | --- | --- | --- | --- | --- | --- | --- | --- | --- | --- | --- |
| **Height0** | 1.000 | 0.956 | 0.739 | 0.127 | 0.238 | 0.739 | 0.738 | 0.115 | 0.090 | 0.388 | 0.332 | 0.212 | -0.193 | -0.220 | 0.341 | 0.148 |
| **StemL0** | -- | 1.000 | 0.617 | 0.174 | 0.286 | 0.753 | 0.668 | 0.227 | 0.031 | 0.384 | 0.353 | 0.185 | -0.163 | -0.126 | 0.266 | 0.228 |
| **Diam0** | -- | -- | 1.000 | 0.159 | -0.140 | 0.441 | 0.834 | -0.030 | 0.069 | 0.215 | 0.127 | 0.356 | -0.062 | -0.270 | 0.241 | -0.314 |
| **Leaves0** | -- | -- | -- | 1.000 | -0.046 | 0.053 | 0.234 | -0.064 | -0.195 | -0.064 | -0.057 | 0.001 | 0.220 | -0.098 | 0.080 | -0.168 |
| **Height:Diam0** | -- | -- | -- | -- | 1.000 | 0.251 | 0.019 | 0.206 | 0.192 | 0.210 | 0.241 | -0.064 | -0.241 | 0.083 | 0.006 | 0.311 |
| **Height2** | -- | -- | -- | -- | -- | 1.000 | 0.679 | 0.374 | 0.305 | 0.734 | 0.696 | 0.289 | -0.195 | -0.048 | 0.277 | 0.521 |
| **Diam2** | -- | -- | -- | -- | -- | -- | 1.000 | 0.163 | 0.207 | 0.456 | 0.394 | 0.416 | -0.125 | -0.174 | 0.195 | -0.200 |
| **Latbranch2** | -- | -- | -- | -- | -- | -- | -- | 1.000 | 0.136 | 0.294 | 0.309 | 0.108 | -0.030 | 0.530 | -0.373 | 0.244 |
| **AgeF** | -- | -- | -- | -- | -- | -- | -- | -- | 1.000 | 0.329 | 0.336 | -0.020 | -0.165 | -0.026 | 0.121 | 0.147 |
| **HeightF** | -- | -- | -- | -- | -- | -- | -- | -- | -- | 1.000 | 0.980 | 0.369 | -0.169 | 0.022 | 0.262 | 0.530 |
| **StemLF** | -- | -- | -- | -- | -- | -- | -- | -- | -- | -- | 1.000 | 0.332 | -0.156 | 0.067 | 0.201 | 0.543 |
| **DiamF** | -- | -- | -- | -- | -- | -- | -- | -- | -- | -- | -- | 1.000 | 0.142 | -0.082 | 0.108 | -0.034 |
| **LeavesF** | -- | -- | -- | -- | -- | -- | -- | -- | -- | -- | -- | -- | 1.000 | 0.082 | -0.174 | -0.122 |
| **Stemtips** | -- | -- | -- | -- | -- | -- | -- | -- | -- | -- | -- | -- | -- | 1.000 | -0.557 | 0.147 |
| **Height:stemtip** | -- | -- | -- | -- | -- | -- | -- | -- | -- | -- | -- | -- | -- | -- | 1.000 | 0.198 |
| **Height:Diam2** | -- | -- | -- | -- | -- | -- | -- | -- | -- | -- | -- | -- | -- | -- | -- | 1.000 |
| **Height0** = Baseline plant height; **StemL0** = baseline stem length; **Diam0** = baseline stem diameter; **Leaves0** = baseline number of leaves below 1 cm; **Height:Diam0** = baseline height to diameter ratio; **Height2** = plant height two months after treatment; **Diam2** = stem diameter two months after treatment; **Latbranch2** = number of lateral branches two months after treatment; **AgeF** = seedling age at end of experiment; **HeightF** = plant height at end of experiment; **StemLF** = stem length at end of experiment; **DiamF** = stem diameter at end of experiment; **LeavesF** = number of leaves below 1 cm at end of experiment; **Stemtips** = number of stem tips; **Height:stemtip** = height to stem tip ratio; **Height:Diam2** = height to diameter ratio two months after treatment. | | | | | | | | | | | | | | | | |

**Table S5:** Results for linear mixed-effects models (LMM) or generalized linear mixed effects models (GLMM) determining how a selection of architectural traits of **surviving tree seedlings** are affected by fire and frost damage. All response variables were measured two months after treatment, except diameter:stem tip ratio which was measured at the end of the experiment. Variance inflation factors (VIF) indicates multicollinearity of predictor variables. R^2^ and model p-values are given in Appendix Table 3 and pairwise comparisons in Appendix Table 6. Significance codes: NS: not significant; *: p<0.05; **: p<0.01; ***: p<0.0001.

| **Response** | **Fixed effects:** | **Estimate** | **Std. error** | **t-value** | **p-value** | **VIF** |
| --- | --- | --- | --- | --- | --- | --- |
| **Plant height**  (LMM) | Intercept | 4.303 | 3.189 | 13.490 | *** | - |
|  | Treatment | - | - | - | *** | 1.922 |
|  | Baseline plant height | 2.847 | 6.007 | 4.740 | *** | 1.559 |
|  | Seedling age | 1.464 | 3.324 | 0.044 | NS | 3.796 |
|  | **Random effects:** | **Variance** | **Std. dev.** |  |  |  |
|  | Species ID | 0.882 | 0.939 |  |  | - |
|  | Residual | 0.924 | 0.961 |  |  | - |
| **Stem diameter**  (LMM) | **Fixed effects:** | **Estimate** | **Std. error** | **t-value** | **p-value** | **VIF** |
|  | Intercept | 1.049 | 0.149 | 7.044 | *** | - |
|  | Treatment | - | - | - | *** | 1.863 |
|  | Baseline diameter | 0.072 | 0.019 | 3.814 | *** | 1.562 |
|  | Seedling age | 0.002 | 0.002 | 0.954 | NS | 3.788 |
|  | **Random effects:** | **Variance** | **Std. dev.** |  |  |  |
|  | Species ID | 0.192 | 0.438 |  |  | - |
|  | Residual | 0.210 | 0.458 |  |  | - |
| **Number of**  **lateral branches**  (GLMM) | **Fixed effects:** | **Estimate** | **Std. error** | **z-value** | **p-value** | **VIF** |
|  | Intercept | 0.488 | 0.268 | 1.821 | NS | - |
|  | Treatment | - | - | - | *** | 2.205 |
|  | Baseline stem length | 0.006 | 0.003 | 2.039 | * | 1.763 |
|  | Seedling age | 0.007 | 0.002 | 3.691 | *** | 4.855 |
|  | **Random effects:** | **Variance** | **Std. dev.** |  |  |  |
|  | Species ID | 0.908 | 0.953 |  |  | - |
|  | **Fixed effects:** | **Estimate** | **Std. error** | **t-value** | **p-value** | **VIF** |
| **Plant height:**  **diameter ratio**  (LMM) | Intercept | 1.431 | 0.123 | 11.596 | *** | - |
|  | Treatment | - | - | - | *** | 1.804 |
|  | Seedling age | 0.003 | 0.001 | 2.280 | * | 3.256 |
|  | **Random effects:** | **Variance** | **Std. dev.** |  |  |  |
|  | Species ID | 0.107 | 0.327 |  |  | - |
|  | Residual | 0.174 | 0.417 |  |  | - |
|  | **Fixed effects:** | **Estimate** | **Std. error** | **t-value** | **p-value** | **VIF** |
| **Plant height:**  **stem tip ratio**  (LMM) | Intercept | 13.707 | 2.491 | 5.502 | *** | - |
|  | Treatment | - | - | - | * | 1.000 |
|  | Seedling age | -0.001 | 0.007 | -0.140 | NS | 1.000 |
|  | **Random effects:** | **Variance** | **Std. dev.** |  |  |  |
|  | Species ID | 9.964 | 3.157 |  |  | - |
|  | Residual | 8.710 | 2.951 |  |  | - |

**Table S6:** Pairwise comparisons for linear mixed-effects models (LMM) or generalized linear mixed-effects models (GLMM) detailed in Appendix Table 4 testing differences in seedling architectural traits between treatments for all survivors. Significance codes: NS: not significant; *: p<0.05; **: p<0.001; ***: p<0.0001.

|  | **Fixed effects:** | **Estimate** | **Std. error** | **t/z-value** | **p-value** |
| --- | --- | --- | --- | --- | --- |
| **Plant height**  (LMM) | Control - Fire | 2.921 | 0.239 | 12.220 | *** |
|  | Control - Frost | 1.763 | 0.445 | 3.960 | *** |
|  | Fire - Frost | -1.158 | 0.294 | -3.942 | *** |
| **Main stem**  **diameter**  (LMM) | Control - Fire | 1.043 | 0.114 | 9.143 | *** |
|  | Control - Frost | 0.833 | 0.210 | 3.963 | *** |
|  | Fire - Frost | -0.210 | 0.134 | -1.568 | NS |
| **Number of**  **lateral branches**  (GLMM) | Control - Fire | 0.804 | 0.150 | 5.353 | *** |
|  | Control - Frost | 0.995 | 0.280 | 3.551 | ** |
|  | Fire - Frost | 0.191 | 0.171 | 1.122 | NS |
| **Plant height:**  **diameter ratio**  (LMM) | Control - Fire | 0.747 | 0.103 | 7.240 | *** |
|  | Control - Frost | 0.809 | 0.185 | 4.382 | *** |
|  | Fire - Frost | 0.061 | 0.115 | 0.535 | NS |
| **Plant height to**  **stem tip ratio**  (LMM) | Control - Fire | 0.5097 | 0.3641 | 1.400 | NS |
|  | Control - Frost | 1.7779 | 0.3483 | 5.104 | *** |
|  | Fire - Frost | 1.2682 | 0.3937 | 3.221 | ** |

**Table S7:** Results for linear mixed-effects models (LMM) or generalized linear mixed effects models (GLMM) determining how a selection of architectural traits of **resprouted tree seedlings** are affected by fire and frost damage. All response variables were measured two months after treatment, except diameter:stem tip ratio which was measured at the end of the experiment. Variance inflation factors (VIF) indicate multicollinearity of predictor variables. R^2^ and model p-values are given in Appendix Table 3. Significance codes: NS: not significant; *: p<0.05; **: p<0.01; ***: p<0.0001.

| **Response** | **Fixed effects:** | **Estimate** | **Std. error** | **t-value** | **p-value** | **VIF** |
| --- | --- | --- | --- | --- | --- | --- |
| **Plant height**  (LMM) | Intercept | 0.405 | 0.298 | 1.361 | NS | - |
|  | Treatment | -0.200 | 0.204 | -0.983 | NS | 3.846 |
|  | Baseline plant height | 0.018 | 0.005 | 4.027 | *** | 1.513 |
|  | Seedling age | 0.004 | 0.002 | 1.891 | NS | 4.101 |
|  | **Random effects:** | **Variance** | **Std. dev.** |  |  |  |
|  | Species ID | 0.115 | 0.339 |  |  | - |
|  | Residual | 0.230 | 0.479 |  |  | - |
| **Stem diameter**  (LMM) | **Fixed effects:** | **Estimate** | **Std. error** | **t-value** | **p-value** | **VIF** |
|  | Intercept | 1.145 | 0.146 | 7.845 | *** | - |
|  | Treatment | -0.029 | 0.050 | -0.575 | NS | 1.008 |
|  | Baseline diameter | 0.050 | 0.011 | 4.426 | *** | 1.008 |
|  | **Random effects:** | **Variance** | **Std. dev.** |  |  |  |
|  | Species ID | 0.216 | 0.465 |  |  | - |
|  | Residual | 0.050 | 0.223 |  |  | - |
| **Number of**  **lateral branches**  (GLMM) | **Fixed effects:** | **Estimate** | **Std. error** | **z-value** | **p-value** | **VIF** |
|  | Intercept | 0.296 | 0.383 | 0.774 | NS |  |
|  | Treatment | -0.212 | 0.241 | -0.878 | NS | 3.765 |
|  | Baseline stem length | 0.014 | 0.005 | 2.668 | ** | 1.418 |
|  | Seedling age | 0.003 | 0.003 | 1.243 | NS | 3.971 |
|  | **Random effects:** | **Variance** | **Std. dev.** |  |  |  |
|  | Species ID | 0.228 | 0.477 |  |  | - |
|  | **Fixed effects:** | **Estimate** | **Std. error** | **t-value** | **p-value** | **VIF** |
| **Plant height:**  **diameter ratio**  (LMM) | Intercept | 0.366 | 0.289 | 1.268 | NS |  |
|  | Treatment | -0.193 | 0.161 | -1.198 | NS | 2.855 |
|  | Seedling age | 0.005 | 0.002 | 2.682 | ** | 2.855 |
|  | **Random effects:** | **Variance** | **Std. dev.** |  |  |  |
|  | Species ID | 0.187 | 0.432 |  |  | - |
|  | Residual | 0.188 | 0.433 |  |  | - |
|  | **Fixed effects:** | **Estimate** | **Std. error** | **t-value** | **p-value** | **VIF** |
| **Plant height:**  **stem tip ratio**  (LMM) | Intercept | 5.133 | 0.622 | 8.249 | *** | - |
|  | Treatment | -0.149 | 0.104 | -1.428 | NS | 1.002 |
|  | Seedling age | 0.0003 | 0.002 | 0.173 | NS | 1.002 |
|  | **Random effects:** | **Variance** | **Std. dev.** |  |  |  |
|  | Species ID | 0.108 | 0.329 |  |  | - |
|  | Residual | 0.231 | 0.481 |  |  | - |
